# Effect of Reading Rehabilitation for Age-Related Macular Degeneration on Cognitive Functioning: Protocol for a Nonrandomized Pre-Post Intervention Study

**DOI:** 10.2196/19931

**Published:** 2021-03-11

**Authors:** Walter Wittich, M Kathleen Pichora-Fuller, Aaron Johnson, Sven Joubert, Eva Kehayia, Vanessa Bachir, Gabrielle Aubin, Atul Jaiswal, Natalie Phillips

**Affiliations:** 1 School of Optometry Université de Montréal Montreal, QC Canada; 2 Institut Nazareth et Louis-Braille du CISSS de la Montérégie-Centre Longueuil, QC Canada; 3 Centre de réadaptation Lethbridge-Layton-Mackay du CIUSSS du Centre-Ouest-de-l’Île-de-Montréal Montreal, QC Canada; 4 Center for Interdisciplinary Rehabilitation Research of Greater Montreal Montreal, QC Canada; 5 Department of Psychology Concordia University Montreal, QC Canada; 6 School of Physical and Occupational Therapy McGill University Montreal, QC Canada; 7 Department of Psychology University of Toronto at Mississauga Mississauga, ON Canada; 8 Department of Psychology Université de Montréal Montreal, QC Canada; 9 Centre de recherche de l'Institut universitaire de gériatrie de Montréal Montreal, QC Canada

**Keywords:** low vision, rehabilitation, cognition, aging, dementia, reading

## Abstract

**Background:**

Age-related vision impairments and dementia both become more prevalent with increasing age. Research into the mechanisms of these conditions has proposed that some of their causes (eg, macular degeneration/glaucoma and Alzheimer’s disease) could be symptoms of an underlying common cause. Research into sensory-cognitive aging has provided data that sensory decline may be linked to the progression of dementia through reduced sensory stimulation. While hearing loss rehabilitation may have a beneficial effect on cognitive functioning, there are no data available on whether low vision rehabilitation, specifically for reading, could have a beneficial effect on cognitive health.

**Objective:**

The research questions are: (1) Does low vision rehabilitation reduce reading effort? (2) If so, does reduced reading effort increase reading activity, and (3) If so, does increased reading activity improve cognitive functioning? The primary objective is to evaluate cognition before, as well as at 6 months and 12 months after, 3 weeks of low vision reading rehabilitation using magnification in individuals with age-related macular degeneration, with or without coexisting hearing impairments. We hypothesize that improvements postrehab will be observed at 6 months and maintained at 12 months for participants with vision loss and less so for those with dual sensory loss. The secondary objective is to correlate participant characteristics with all cognitive outcomes to identify which may play an important role in reading rehabilitation.

**Methods:**

We employ a quasiexperimental approach (nonrandomized, pre-post intervention study). A 3x3 design (3 groups x 3 time points) allows us to examine whether cognitive performance will change before and after 6 months and 12 months of a low vision reading intervention, when comparing 75 low vision and 75 dual sensory impaired (vision & hearing) participants to 75 age-matched healthy controls. The study includes outcome measures of vision (eg, reading acuity and speed), cognition (eg, short-term and long-term memory, processing speed), participant descriptors, demographics, and clinical data (eg, speech perception in noise, mental health).

**Results:**

The study has received approval, and recruitment began on April 24, 2019. As of March 4, 2021, 38 low vision and 7 control participants have been enrolled. Lockdown forced a pause in recruitment, which will recommence once the COVID-19 crisis has reached a point where face-to-face data collection with older adults becomes feasible again.

**Conclusions:**

Evidence of protective effects caused by reading rehabilitation will have a considerable impact on the vision rehabilitation community and their clients as well as all professionals involved in the care of older adults with or without dementia. If we demonstrate that reading rehabilitation has a beneficial effect on cognition, the demand for rehabilitation services will increase, potentially preventing cognitive decline across groups of older adults at risk of developing macular degeneration.

**Trial Registration:**

ClinicalTrials.gov NCT04276610; Unique Protocol ID: CRIR-1284-1217; https://clinicaltrials.gov/ct2/show/NCT04276610

**International Registered Report Identifier (IRRID):**

DERR1-10.2196/19931

## Introduction

### Sensory and Cognitive Loss—An International, National, and Local Priority

Prevention and treatment of cognitive impairments in the aging population have become priorities for stakeholders in health care research around the globe. In Canada, the Senate Report on the need for a Canadian Dementia Strategy was released in 2017 [1], and resulting recommendations started to be made public in 2019 [2]. For example, the importance of vision and hearing is mentioned in order to promote and enable early diagnosis, with the goals of increasing quality of life and improving social connectedness, belonging, and purpose. These initiatives specifically refer to the importance of vision and hearing health research. Internationally, recent publications in The Lancet [3,4] described dementia as the most challenging threat to population health in our century and pointed out that hearing loss may be the largest potentially modifiable risk factor for cognitive impairments. While low vision rehabilitation (eg, magnification strategies and reading rehabilitation) may serve as a potential prevention strategy for cognitive decline, this possibility is notably absent from this review. There is simply a lack of evidence from longitudinal or intervention studies regarding the links between visual loss and cognitive impairments [5]. Some researchers have indicated that improvement of vision through cataract surgery has resulted in improved scores on attention, orientation, memory, language, visual perceptual, and visuospatial skills as measured by the Addenbrooke’s Cognitive Examination or the Revised Hasegawa’s dementia scale [6,7]; however, improvements in global scores across these cognitive domains did not replicate when using the Telephone Interview of Cognitive Status [8,9]. This may in part be explained by the fact that some of these researchers chose to utilize cognitive measures [10] that contain items that require vision without any adaptations to confirm the visibility of these test items. Others utilized nonvisual measures of cognition [11,12], thereby avoiding the possible influence of improved vision masking as improved cognition on visual test items. Apart from medical interventions such as cataract surgery, the main technique to improve visual input for reading in the presence of low vision has been magnification; however, vision rehabilitation has never been systematically evaluated using cognitive outcome measures.

### Comorbidity of Ocular Disease and Cognitive Impairments

There is a growing body of evidence linking age-related eye diseases such as age-related macular degeneration (AMD) with changes in cognitive functioning and cognitive impairments due to Alzheimer’s disease (AD) [13-17]. The prevalences of AMD and AD increase with increasing age [18-21]; both conditions share many risk factors (eg, smoking, obesity, age, and unhealthy diet [22]), yet their comorbidity is higher than what would be expected if they were independent of each other [23-25]. The anatomical changes observed in both AMD (eg, drusen development in the retina) and AD (eg, formation of plaques in the brain) are possible symptoms of a common underlying disease mechanism within the central nervous system. Specifically, the buildup of beta amyloid found in plaques and drusen could indicate a common pathogenesis for both diseases [26-28]. Furthermore, declines in visual and cognitive functioning are correlated [29-31]; for example, higher cognitive function scores on the Mini-Mental State Examination (MMSE) were associated with better best-corrected visual acuity [29], when the visual items on this cognitive screening test are either included or excluded [31]. Declining scores on a modified and expanded version of the MMSE correlated with declines in visual acuity, contrast sensitivity, and stereo acuity impairments over 9 years in a population-based study of community-dwelling, highly physically functioning older adults [30]. It is less clear, however, whether these declines can be modified with vision interventions, given that the MMSE is a screening tool covering multiple aspects of cognition but none at great depth. There is some evidence that individuals who read frequently are at reduced risk of developing cognitive impairments [32]. In addition, it is not surprising that there is overlap between the behavioral aspects of AMD and AD; for example, social disengagement and functional impairments in activities of daily living may be present as a result of either or both conditions.

### Low Vision Makes Reading More Effortful

The Framework for Understanding Effortful Listening has been used to illustrate how listeners with a hearing impairment allocate more cognitive resources in challenging conditions (such as when an individual has hearing loss or there is noise) [33]. Similarly, reading becomes more effortful in the presence of central visual impairment or when visual input is suboptimal. For example, AMD generally causes a reduction in visual acuity, making it necessary for persons living with AMD to utilize magnification in order to read with peripheral retina that is still intact [34,35]. Given that fixation in the periphery is less stable [36], more concentration and effort are required to read magnified text with a peripheral retinal locus [37,38]. Effortful viewing or reading explains why the presence of central visual impairment (eg, the presence of central scotoma and resulting drop in visual acuity) has repeatedly been correlated with reduced reading speed as low as 20-40 words per minute [39], whereby poorer fixation stability has been associated with slower reading speeds [40]. Increased cognitive load leads to a decrease in the attentional visual window [41]. As individuals with vision loss find reading more effortful, thus experiencing a higher cognitive load, their attentional window should shrink. Processing of visual information in the retinal periphery (as is necessary in persons living with AMD) is slower and not as efficient as in the macular region [42], in both younger and older observers [43]. In addition, low vision affects eye movements during reading, thereby further shrinking the perceptual window where letters are processed, likely due to the increase in cognitive demand [44]. Importantly, when some cognitive resources are diverted to this reading effort, remaining cognitive resources may be insufficient for readers to rapidly or accurately process (comprehend or remember) the information that was seen or read.

### Low Vision Rehabilitation Reduces Reading Effort

Difficulty while reading is the most common functional complaint in persons with low vision, and improving the ability to read is often the main purpose of vision rehabilitation interventions [45]. The effectiveness of low vision rehabilitation has been demonstrated repeatedly [46,47], specifically for reading [48]. A systematic review confirmed that there is strong evidence that low vision reading rehabilitation services improve reading ability overall [49]. Both magnification devices (eg, handheld magnifiers, closed-circuit televisions, or zoom functions on an iPad [50,51]) and large print appear to be equally effective [52]. Increasing reading performance (eg, increasing reading speed at decreased print size or improving comprehension) is frequently the main target for improvement during rehabilitation [49,53]. It has been used as the primary outcome measure in recent clinical trials demonstrating the effectiveness of low vision treatments [49,53]. Individuals living with low vision can be trained to use either closed-circuit television video magnifiers or mechanical magnification devices (eg, handheld magnifiers, telescopes) to help improve reading performance by up to 200% [54]. One central underlying goal of low vision rehabilitation is to reduce the effort that is required to accomplish visual tasks in the presence of a visual impairment [55]. Measuring this effort (or its reduction) can be done subjectively by asking participants whether they perceive less effort during completion of the task. However, there are also a variety of observable variables that can be used as indicators of reduced reading effort. They include improved reading speed (eg, reading becomes faster as it becomes easier) and improved reading comprehension (eg, less effort liberates cognitive resources for processing and retention [33]). Interestingly, the findings for reading comprehension in the presence of low vision are mixed [56] insofar as reading speed (as an indicator of effort), scotoma size, and visual acuity all influence comprehension. However, most studies on low vision reading have small sample sizes and are underpowered. Therefore, it is hard to control for factors such as scotoma size or acuity impairment, a problem we hope to overcome in our study by recruiting a larger number of participants than is generally the case in studies on low vision and reading.

### The Link Between Reading and Cognition

Reading is a complex process that involves bottom-up visual processing to enable grapheme (ie, letter) recognition that in turn enables grapheme-to-phoneme conversion, leading to word recognition and the identification of morphosemantic, syntactic, and pragmatic features of lexical items that are ultimately used in the comprehension of sentences and discourse such as stories [57]. Because reading is subserved by a number of cognitive processes, including attention, long-term memory, and working memory, there is a symbiotic relationship between reading and cognitive processing as has been documented extensively in the psycholinguistic/neurolinguistic and brain imaging literature [58,59]. Notably, a number of studies has shown that engaging in high-level cognitive activities, such as reading text, appears to preserve cognition in aging adults. The frequency of participation in activities that are mentally stimulating, such as reading, is associated with lower risk of incident AD [59-61]. Researchers have also linked reading and engaging in higher-level cognitive activities with increased cognitive reserve, that in turn is associated with more tolerance of AD pathology and stimulation of brain plasticity [62-65].

### Does Reduced Reading Effort Lead to Improved Cognition?

We previously found a positive correlation between reading speed and higher scores on the Montreal Cognitive Assessment (MoCA) in persons with AMD [66,67]. Given the information reviewed in the previous sections, the logical direction for our investigation into the functional connection between low vision and cognition is to examine the possible effects of reading rehabilitation on the cognitive abilities of individuals undergoing low vision rehabilitation. In order to disentangle the effects of visual and cognitive impairments in older adults, members of this research team have adapted cognitive tests so they can be administered to individuals with low vision [68,69]. In parallel, we are also in the process of developing a vision-screening test that can be administered to individuals with various levels of cognitive impairment [70,71]. This investigation will guide our future research efforts into the improvement of service provision in low vision rehabilitation for the purpose of increasing the independence and cognitive health of older adults living with low vision.

### Objectives and Hypotheses

The overall goal of the study is to demonstrate that low vision rehabilitation will improve reading, which, in turn, will improve cognition.

The primary objective is to measure global changes in reading ability and cognitive functioning before and after 6 months and 12 months of reading rehabilitation. Hypothesis 1 is that improvements relative to pretreatment performance will be observed at 6 months and maintained at 12 months for participants with vision loss only and less so for those with dual sensory impairment (DSI). Control participants are expected to have stable performance and outperform both groups who receive reading rehabilitation. The global reading outcomes are reading speed, reading comprehension, and subjective perception of reading effort.

The global measure of cognition is the MoCA.

The secondary objective is to explore changes in more specific subdomains of reading ability and cognitive functioning. Hypothesis 2 is that improvements relative to pretreatment performance will be observed at 6 months and maintained at 12 months for participants with vision loss only and less so for those with DSI. Control participants are expected to have stable performance and outperform both groups who receive reading rehabilitation. The specific reading outcomes are reading acuity, critical print size, reading accuracy, and subjective reports of changes in reading habits.

The specific measures of cognition are episodic learning; memory encoding, storage, and retrieval; attention, speed, and mental flexibility; and semantic fluency.

Hypothesis 3 is that improved reading behavior will be correlated with improved cognitive functioning across all 3 groups, with the strongest relationship being in the AMD-only group. Such correlations will be observed across the global as well as the specific measures.

The tertiary objective is to explore factors that may influence the cognitive benefits of reading rehabilitation. Hypothesis 4 is that individual differences in the association between improved reading behavior and cognitive functioning may be related to participant characteristics such as demographic variables, hearing impairment severity, and mental health.

### Study Design

We present this protocol following the Standard Protocol Items: Recommendations for Interventional Trials (SPIRIT) guidelines [72]. An overview of the trial registration data is provided in Table 1. We decided on a 3-arm, quasiexperimental, repeated-measures design (nonrandomized, pre-post intervention study) [73], whereby participants act as their own comparison across time points. Given the prevalence of hearing loss among older adults, we decided to include both patients with AMD who present with normal hearing [74,75] and those who experience both vision and hearing loss (DSI). We decided to add an age-matched control group with healthy vision and hearing in order to observe the size of possible practice effects and record general variability in the measures, given the expected advanced age of our participants. The rehabilitation center partners on this study generally provide reading rehabilitation within 3 months of the initial optometric assessment. Therefore, we chose 6 and 12 months as suitable follow-up time points. After 6 months, the initial interventions will be completed, and participants will have had 3 months to engage in reading postintervention. Overall, a design with 3 groups (AMD-only, DSI, comparison) x 3 test times (preintervention, 6 months, 12 months) is planned to allow us to examine whether cognitive performance will change over time and if the degree of change and the final performance on outcome measures will differ across groups.

**Table 1:** Trial registration data.

Data category	Information
Primary registry and trial identifying number	ClinicalTrials.gov ID: NCT04276610
Date of registration in primary registry	February 19, 2020
Secondary identifying numbers	CRIR-1284-1217
Source(s) of monetary or material support Primary sponsor	Fonds de recherche Quebec - Santé & Turmel Foundation (years 1 & 2); Canadian Institutes of Health Research Project Grant: Patient-Oriented Research Priority Announcement (year 3)
Contact for public queries	Walter.wittich@umontreal.ca
Contact for scientific queries	Walter.wittich@umontreal.ca; natalie.phillips@concordia.ca
Public & scientific title	Words on the Brain: Can Reading Rehabilitation for Age-Related Vision Impairment Improve Cognitive Functioning?
Countries of recruitment	Canada
Health condition(s) or problem(s) studied	Age-related macular degeneration Dementia
Intervention(s)	Behavioral: Low Vision Reading Rehabilitation
Study type	Interventional clinical trial, nonrandomized, parallel assignment
Date of first enrollment	April 24, 2019
Target sample size	225
Recruitment status	Recruiting

## Methods

### Study Setting

The study will be conducted at 2 partnering vision rehabilitation centers in the Montreal area, the Centre de réadaptation Lethbridge-Layton-Mackay du Centre intégré universitaire de santé et de services sociaux du Centre-Ouest- de-l’Île-de-Montréal and the Institut Nazareth et Louis-Braille du Centre intégré de santé et de services sociaux de la Montérégie-Centre. Both sites are part of the Center for Interdisciplinary Rehabilitation Research of Greater Montreal and provide government-funded rehabilitation services, free of charge for eligible residents of Quebec, Canada.

### Eligibility Criteria

#### Inclusion Criteria

Participants in the intervention groups (AMD-only and DSI) are required to have a primary diagnosis of AMD (any type, as drusen-containing beta amyloid are found in all types of AMD [26-28]) as confirmed by the ophthalmologist or optometrist who referred the individual to the vision rehabilitation centers. They must be able to benefit from a magnification intervention for the purpose of improving their ability to read, according to the clinical judgment of their rehabilitation professionals who coconstruct their personalized intervention plans. All participants need to be able to communicate in either English or French (their choice of dominant language) and have a distance visual acuity in the better eye of 20/60 or less with best standard refraction, according to the admission criteria for eligibility for rehabilitation services in Quebec [76]. Individuals in the healthy control group are required to have age-normal hearing and vision (visual acuity better than 20/40 in the better eye, no diagnosis of visual impairment in the last 12 months) [77]. In line with the hearing impairment categorizations used in Quebec [78], participants with unaided pure-tone averages (PTAs) across .5, 1, 2, and 4 kHz in the better ear of 40 decibel hearing level (dB HL) or less will be considered to have no or mild hearing impairment. Those with PTAs between 41 and 79 dB HL will be considered participants with moderate to severe hearing impairment and will be allocated to the DSI group. We will also recruit 75 age-matched older adults without visual impairment.

#### Exclusion Criteria

Participants cannot currently be undergoing any medical treatment for their AMD (eg, antivascular endothelial growth factor therapy injections). They must have sufficient residual vision to benefit from magnification for the purpose of reading printed paragraphs (visual acuity in the better eye of 20/400 or better). Based on past experience with participant recruitment over the phone, recruitment success over the phone is low with individuals living with more severe degrees of hearing impairment. Therefore, those whose file information indicates an audiogram with an unaided PTA ≥80 dB HL in their better ear will not be approached for recruitment [79]. In order to facilitate the administration of informed written consent through the research assistants and to focus recruitment on older adults with the necessary cognitive capacities to complete a somewhat lengthy protocol, individuals whose file information indicates a diagnosis of an advanced cognitive impairment such as AD will not be approached for recruitment. Participants whose total score on the MoCA is below 18 or whose score on the blind MoCA is below 10 will not be included in the study, because our clinical and research experience [80] has indicated that individuals with scores at that level are likely too cognitively impaired to complete a research protocol with this level of complexity. At the initial assessment, both the PTA and MoCA scores will be used to determine if participants continue further in the study.

### Intervention

Rehabilitation services at the partner centers will deliver the low vision evaluation and reading rehabilitation intervention, as regulated by the Quebec Ministry of Health. Typically, within 3 months of their initial low vision rehabilitation assessment, individuals will receive most of the recommended interventions. The clinical staff and rehabilitation professionals at either rehabilitation center decide which components of the full complement of services are suited for each participant. The 2 centers offer comparable services, including the provision of assistive devices and services, as regulated by the Quebec Health Insurance Program. These services are similar to those provided within the Blind Rehab Centers of the Veterans Affairs service in the United States [46,47]. They include, but are not limited to, a full optometric exam to determine functional vision, including refraction and the prescription of appropriate near and distance glasses and optical devices; an assessment by a low vision therapist or occupational therapist to establish the participant’s functional priorities and rehabilitation goals; and the provision of handheld optical magnification devices, electronic nonoptical magnification devices (eg, portable or tabletop closed-circuit TVs), or computer software for screen content magnification (eg, ZoomText). All devices are provided free of cost for the individual and with appropriate training and follow-up sessions at home within 3 months of the initial intervention, as required. The rehabilitation professionals may perform a systematic lighting assessment in the participants’ homes [81-83] and may make specific lighting recommendations that are intended to improve their reading ability. In addition, participants will have access to referral services such as orientation and mobility training (for independent travel) or registration with an adapted adult day center for individuals with sensory loss [80]. These centers provide access to psychosocial services, counsellors, social workers, or other mental health professionals. The provision of assistive devices for magnification and reading is generally linked to a follow-up visit in the individual’s home 3 weeks after the initial rehabilitation appointment. At this time, rehabilitation professionals observe the use of the devices and strategies in the environment where the participant lives. It is at this point that the professionals together with the participants decide whether the devices are useful and are assigned as a permanent loan. Should additional needs emerge, the participant is at liberty to contact the rehabilitation center at any time to initiate a new service episode. The rehabilitation professionals record all aspects of this intervention in the rehabilitation files, to which the research team will have access during and at the end of the 12-month study period.

### Outcomes

#### Test Administration

The order of test administration begins with the primary (MoCA, Minnesota Low Vision Reading Test, International Reading Speed Test, reading habits questionnaire) followed by the secondary and then the participant characteristics measures; should a participant be unable to complete any one of the measures due to its complexity or to fatigue, the experimenter moves on to the next test, using personal judgment as to the participant’s level of fatigue. Participants are encouraged to take as many breaks as they desire in order to facilitate completion of the maximum number of tests possible. Incomplete tests will not be scored; however, the number of completed tests at each testing session will be compared. Note that cognitive test administration can be adjusted to make instructions audible for persons with hearing loss. Ambient lighting at home in the room where participants generally read and where the tests are administered will be measured using a Digital Illuminance Meter (model LX1330B, Dr. Meter, Union City, CA).

#### Cognition Outcome Measures

All cognitive measures are administered in the auditory domain and thus will not be affected by a visual impairment. Therefore, should performance on the cognitive measures improve following the vision rehabilitation program, the improvement should be attributable to the benefit of increased visual function on cognitive stimulation as opposed to merely being due to improved perception of the stimuli involved in the tests. It is reasonable to expect improvement on the chosen cognitive measures as a result of the reading intervention and within the timeframe of the study. Notably, the chosen measures assess cognitive domains that have been shown to predict everyday function in older adults: episodic learning and memory [84], processing speed [85], and working memory [86].

General cognition will be measured using the MoCA [87] or its adapted version for persons with visual impairment (MoCA-Blind [68]). Should participants be unable to complete the visual items of this test because their vision is too severely impaired, the adapted version will be administered. The measure covers aspects of executive functioning, memory, language, abstraction, and orientation in time and space. Scoring ranges from 0 to 30 (MoCA) or 0 to 22 (MoCA-Blind), with higher scores indicating better performance. A score of 26 on the MoCA or 18 on the MoCA-Blind is the cut-off indicating that an individual may be at risk for having mild cognitive impairments [68,87]. The MoCA has good psychometric properties (internal reliability: a=.83; test-retest reliability: r=.92; validity: r=.87). The MoCA is available in multiple equivalent versions and in multiple languages. One of the 3 available versions of the MoCA will be assigned randomly for each participant at the first session in order to reduce practice effects. The other 2 versions will be used for sessions 2 and 3.

Auditory episodic learning and memory will be assessed using the Rey Auditory Verbal Learning Test (RAVLT) [88]. This test evaluates memory encoding, storage, and retrieval. Its psychometric properties indicate an internal reliability of .90 and test-retest variability ranging from r=.60 to r=.70 [88]. Both parts of the RAVLT (acquisition, as well as delayed recall and recognition) will be used. In the acquisition part of the test, the experimenter reads a list of 15 words that the participant needs to repeat immediately afterwards. The experimenter reads the list 5 times, and after each time, the participant repeats the words remembered from the same 15-word list. The experimenter then reads a second list of words once in order to create interference. The participants are asked to say as many words as they can remember from this list. After the interference task, the participant is asked to recall as many words as possible from the first list. After a 20-minute break, the participant is asked to recall as many words as possible from the first list. Then, the experimenter reads a list of 50 words and asks the participant to identify which of these words were contained in the first list (recognition task). Acquisition (ie, number of words recalled in the first 5 trials), delayed recall (ie, the number of words recalled after the 20-minute interval), and recognition (ie, number of words correctly identified in a 50-word list) are the components of the RAVLT. Higher scores indicate better performance.

The Oral Trail Making Test parts A & B [89] evaluate sequential set-shifting without the motor and visual demands of its written counterpart, thereby being ideally adapted for persons with visual impairment [88]. This test assesses attention, speed, and mental flexibility. Validity has been reported at .68 for Part A and .72 for Part B [88]. In part A of the test, participants are asked to count from 1 to 25 as fast as possible while maintaining intelligibility. In part B, they are asked to count to 13 by alternating a number and a letter in numerical and alphabetical order. The outcome measure is the time, in seconds, participants take to complete the task, with lower scores indicating better performance.

Semantic fluency describes the ability to successfully retrieve specific information within a time limit. Fluency will be measured with the letters F, A, and S, as well as by animal naming [90]. Reported psychometric properties include internal reliability of r=.83, test-retest reliability of r=.74, and validity ranging from r=.85 to r=.94 [88]. Specifically, the participant is asked to generate as many words as possible starting with the letters F, A, and S, as well as to name as many animals as possible, all within 1 minute. The instructions are to avoid repetitions, proper names, or slang. Higher scores indicate better performance.

#### Reading Outcome Measures

The secondary measures pertain to reading, including past and present reading habits, reading acuity, reading speed, and reading comprehension. These measures will demonstrate if there are direct benefits from low vision rehabilitation on reading. Multiple tests were chosen in order to capture various aspects of reading under different conditions.

To subjectively assess reading and reading effort, we will administer a questionnaire about reading habits that was developed specifically to evaluate the extent to and frequency with which an individual engages in reading activities during activities of daily living, including reading for entertainment or education. Our team has previously employed this measure [50]. It includes questions on language background (eg, first language, language proficiency), assessed self-reported proficiency in reading (1=no ability, 5=fluent ability), reading habits before and after onset of low vision (eg, frequency, enjoyment, type of reading), and enjoyment of reading. In order to evaluate the subjective experience of reading effort, participants will be asked: “When considering your vision, is reading easy, somewhat difficult, very difficult, or impossible?”

To objectively measure reading performance of short individual sentences, participants will read the English or French version of the Minnesota Low Vision Reading Test chart [91], a clinical assessment chart that allows for the measurement of reading acuity (smallest print read), reading speed (in words per minute), critical print size (smallest print at which reading speed is still optimal), and the Reading Accessibility Index [92], which considers reading ability over a range of print sizes. Its test-retest reliability has been reported at r=.88 [92-94].

In order to measure sustained reading of text in paragraph format, participants will be asked to read English or French paragraphs from the International Reading Speed Test [95]. This measure includes reading comprehension questions that have previously been developed and used in our lab in the context of a low vision reading evaluation using the iPad as a magnification device [50]. Its internal reliability has been reported ranging from a=.77 to a=.93 [95,96].

Finally, we will administer a semantic judgment task using a calibrated computer display, allowing us to capture reaction time and response congruency during a sentence-reading task. Here, participants must first read a sentence (prime) and then see a word (target). Their task is to determine if the target word completes the sentence in a way that makes sense or not. An equal number of congruent and incongruent sentences is included, and dependent variables are accuracy and speed. Sentences chosen for this task all have established completion and Cloze probability norms ([97] for the French and [98] English sentences). During this task, we obtain measures on response speed and accuracy, allowing us to evaluate reading speed, vocabulary comprehension, and ability to judge semantic congruency.

#### Participant Characteristics

Hearing ability will be assessed as part of the protocol because hearing has been identified as a risk factor for cognitive impairments [4]. We designed the cognitive assessment procedure in such a way that vision is not required for the administration of the auditory testing materials. We will document the ambient sound levels during testing using the Decibel X app by SkyPaw Co Ltd (Hanoi, Vietnam), because testing may be conducted in participants’ homes. Ambient noise levels will be used to statistically explore potential noise effects on hearing thresholds. For individuals who experience difficulties hearing the experimental protocol instructions, a Williams Sound Pocketalker (Eden Prairie, MN [99]) will be used to provide amplification.

The integrity of the ear canal will be inspected using the Welch Allyn 22820 PocketScope Otoscope, and the presence of any abnormalities (eg, impacted cerumen, collapsing canals) will be noted.

Participants for whom a recent audiogram is not available in the rehabilitation file will complete a pure-tone audiogram administered using a portable audiometer (Maico MA41, Berlin, Germany, from GénieAudio, Laval, Quebec) with Radioear DD45 earphones. We will use the audiometric results to calculate the PTA dB HL threshold across 0.5, 1, 2, and 4 kHz in each ear. The PTA will be used to determine which participants become part of the DSI group.

Speech-in-noise thresholds will be measured using the Canadian Digit Triplet Test, validated both in Canadian English and French [100,101]. During this test, participants listen under headphones to triplets of spoken digits (eg, 2, 9, 5) presented in the presence of matched speech-spectrum background noise. The listener can respond nonverbally by entering the digits that have been heard on a numeric keypad or verbally so the tester can enter the responses on the keypad. The Canadian Digit Triplet Test uses an adaptive procedure to compute a speech reception threshold in noise, defined as the signal-to-noise ratio at which the triplets are recognized 50% of the time. There is a high level of consistency across the English and French versions of the test [101]. The more negative the score, the more noise the listener can tolerate.

Participants will complete the Hearing Handicap Inventory for the Elderly questionnaire [102,103] and answer 3 individual questions about hearing ability, which assess their perception of difficulties with activities of daily living that require hearing [102]. These hearing assessments are the same as those that have been used or are planned for the Canadian Longitudinal Study on Aging [104], thereby allowing us to compare our intervention findings to national population data. The Hearing Handicap Inventory for the Elderly explores self-reported situational and emotional hearing abilities and has a reliability coefficient ranging from .88 to .95 and test-retest reliability of r=.84 [102,105].

The onset of age-related vision loss due to AMD is often accompanied by a multitude of emotional responses that can potentially interfere with the success of low vision rehabilitation [106,107]. Therefore, the Depression, Anxiety and Stress Scale [108] will be included in the protocol in order to identify individuals whose rehabilitation outcomes might be influenced by their psychological state; however, they are not excluded depending on this measure. This 21-item questionnaire shows excellent psychometric properties, with an internal reliability ranging from a=.86 to a=.90 and has been validated in both English and French [109,110].

As part of any low vision exam, the eye care professionals within each rehabilitation center record key variables in the rehabilitation charts. These data will be available for extraction by the research team as described in the study consent and will be extracted at the beginning and end of the study. They include, but are not limited to, diagnoses (ocular and otherwise), monocular and binocular visual acuity (distance vision using ETDRS [111] and contrast sensitivity [Mars chart] [112]), visual field diameter (Octopus perimeter), type and duration of rehabilitation services provided (eg, computer rehabilitation, orientation & mobility services), and type of assistive devices that were prescribed and provided (please note that these devices are provided at no cost to the individual through Quebec Health Insurance/Régie de l'assurance maladie du Québec; therefore, income is not a barrier to device use). The file contains information about the type, frequency, and intensity of vision rehabilitation strategies that were implemented and trained (eg, provision of improved lighting, strategies to read while placing materials on reading stands at the appropriate distance).

In addition, the file contains basic demographic information (eg, presenting gender, age, living situation), as well as a record of all parallel services that were accessed (eg, participation in the day center service, provision of hearing rehabilitation). These clinical variables will be available to the research team for statistical control and analysis.

### Participant Timeline

**Figure 1:**
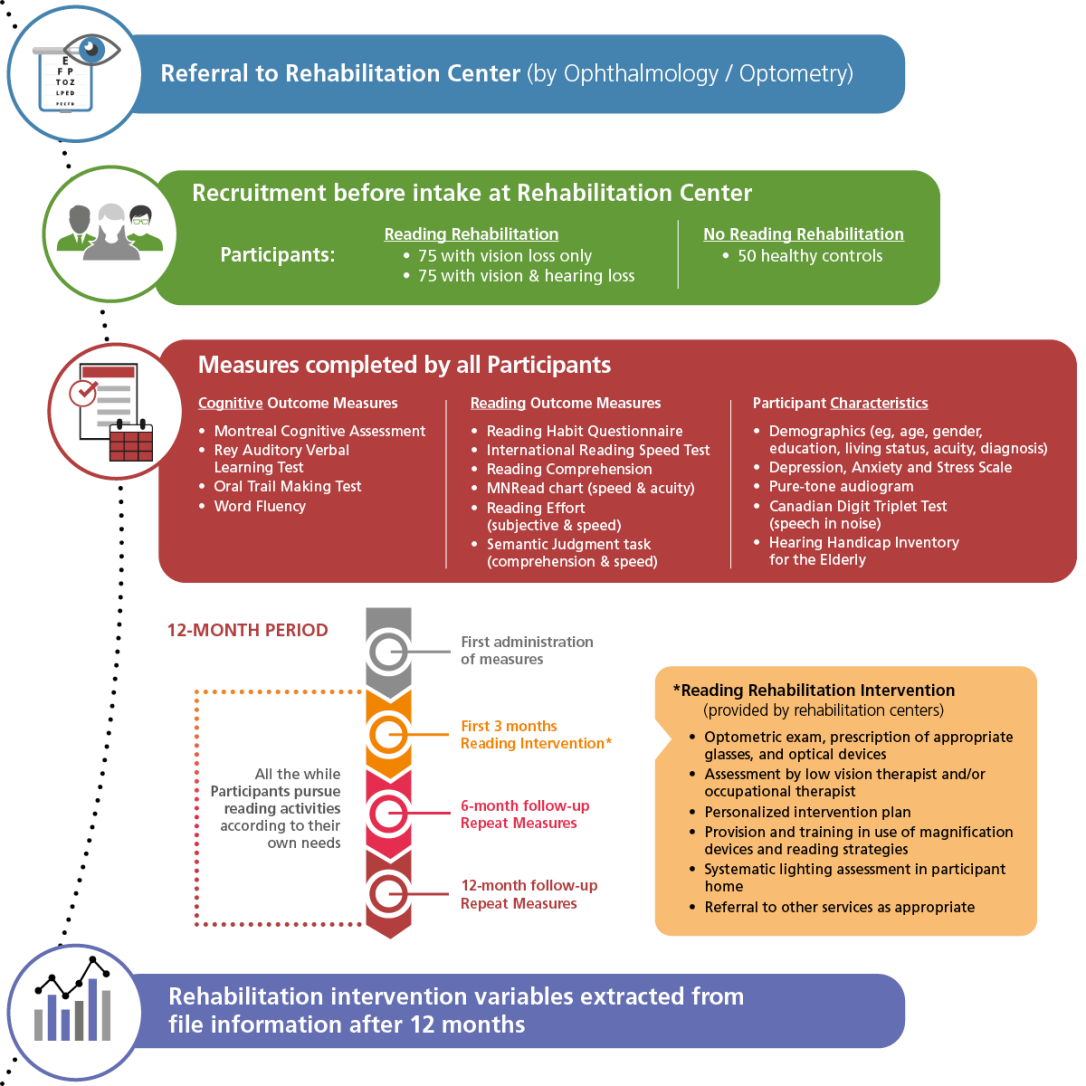
Overview of the study structure, timeline, and variables to be measured.

**Figure 2:**
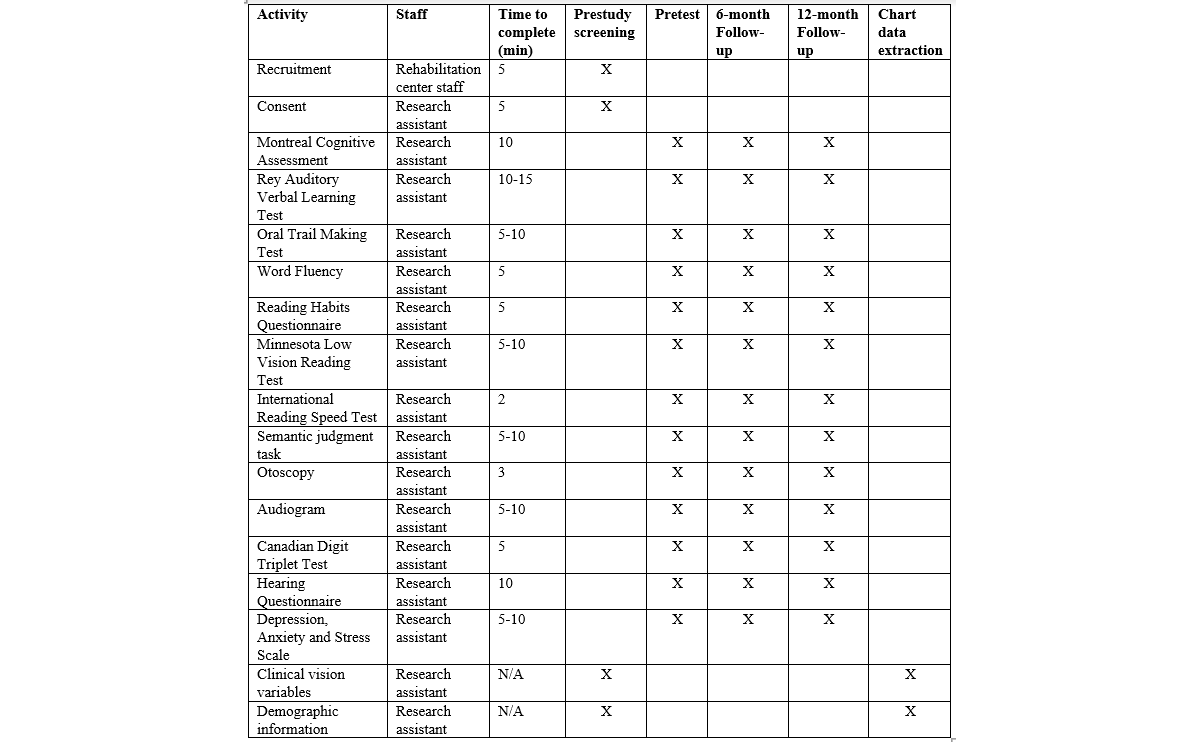
Protocol schedule and procedures. N/A: not applicable.

Figure 1 illustrates the structure and timeline of the study. After intake, but before intervention begins, participants will complete the initial administration of all assessment measures. At 6 months and 12 months after the initial rehabilitation appointment, the research assistants will meet with each participant (at home for rehabilitation participants, in the lab with control participants) to repeat the administration of all measures. After the 12-month follow-up, data will be extracted from the rehabilitation charts detailing all rehabilitation interventions. A detailed overview of this process is presented in Figure 2.

### Sample Size

The power analysis for this study is centered around the MoCA, the primary outcome measure of cognition. We are only aware of 1 study reporting MoCA scores (in its blind version) after a vision rehabilitation intervention, whereby 21 older adults participated in a program evaluation [80]. This study, however, contained reading rehabilitation in addition to various other interventions (eg, orientation and mobility, social engagement in an adapted day center); therefore, the reported effect size of *d*=0.794 (*η^2^* equivalent=0.136) after 6 months likely overestimates the outcomes we would expect in our study design. To be conservative, we halved this expected effect size for our power estimate. Please note that our control group is primarily used to examine possible practice effects and to observe variability on the measures in a cohort with potentially high age. We based our power analysis on a 3x3 (group x time point) within-between design for analysis of variance. Using gPower [113], a minimum sample size of n=81 will allow us to detect small effect sizes of .20 or greater, with an alpha of .05 and a desired power of .95. We also expect an attrition rate of around 10% at each time point based on our previous research with older, visually impaired adults [114,115]. We plan to include covariates in the analyses that will be identified from the analyses linked to the tertiary objective exploring factors that may influence the cognitive benefits of reading rehabilitation. The sample will need to be sufficiently large for these variables to be normally distributed in each group. Therefore, a sample goal of 225 (150 participants with sensory loss, 75 controls) should be sufficient for our protocol. This goal is feasible given an average of 3000 new referrals to rehabilitation in Montreal per year.

### Recruitment

Our partner organizations will conduct recruitment for participants in the intervention groups (AMD-only and DSI) as part of their responsibility on the research team. We will recruit control participants through staff of the partner organizations, from family members of participants, as well as through open advertisements in public media and communications to senior groups. In addition, we have access to the Banque des participants of the Centre de recherche institut universitaire de gériatrie de Montreal (n>1000). Using this database, we can match our control participants to the intervention participants on variables such as age (by decade), presenting gender, education, and other potential variables of interest, such as MoCA score. Our pilot study [66,67] indicated that we will be able to recruit at least 3 AMD or DSI participants per week, making the recruitment target goal feasible to achieve within 18 months.

### Data Collection Methods

Data will be collected by 4 trained research assistants (2 teams of 2 people). The composition of the pairs who work together to collect the data will continuously rotate in order to facilitate harmonization among the teams and ensure consistency and accuracy in the administration of the measures. It will not be possible to conceal group membership from the research assistants because the administration of some of the measures will determine if participants are considered to have a hearing impairment. In addition, control participants will be identifiable because they are not visually impaired. However, the teams will be independent of the administration of the intervention, which will be provided by rehabilitation professionals outside of the research team. Administration of the measures will be audio-recorded throughout several of the tasks to ensure the highest level of precision and quality control for data entry and to improve testing efficiency and reduce testing time. We offer participants the choice of coming to the lab space at either rehabilitation center or receiving a home visit for data collection. Therefore, we developed our protocol to be easily portable such that the necessary measures and materials can be transported wherever needed by the research team using a wheeled carry-on suitcase.

### Data Management

In line with the data management requirements of the Institutional Review Board that approved this protocol and the recommendations by Michener for data management [116], all data will be anonymized and entered in a central password-protected Excel file that will be stored on the encrypted server of the Université de Montréal. Data integrity and quality control will be enforced through random spot-checking by a second member of the team. The data will be accessible for the period of the study approval, plus an additional 5 years thereafter, at which point all electronic and paper files will be destroyed.

### Statistical Methods

#### Planned Analyses

Table 2 provides an overview of the planned analyses. We will examine change in the primary outcome measures (ie, MoCA, reading speed, reading comprehension, subjective report of effort) from baseline over time using generalized linear mixed-effect model analysis employing the *lme4* package [117] and the *bobyca* optimizer in RStudio [118]. For all primary outcomes, we will assess normality using a visual inspection of the quantile-quantile plot and the Shapiro-Wilk test using the function *shapiro.test.*

In comparison to traditional parametric statistics (ie, analyses of variance), mixed-effect models are relatively robust to violations of normality [117]. Generalized linear mixed-effect model analysis can accommodate nonnormally distributed responses or dependent variables and categorical data via their variety of family functions. Each measure will be specified as a continuous dependent variable and examined as a *gamma* model (if outcome measure data are skewed) or *gaussian* model (if data are normally distributed) in the family argument of the *glmer* function as a function of the 2 categorical predictors: group (ie, low vision, DSI, control) and time of testing (ie, before, 6 months after, and 12 months after reading rehabilitation), and their interaction. All generalized linear mixed-effect model analyses will include the maximal random effects structure justified by the experimental design [119]. They will include all main effects and interactions of our 2 predictors, group and time of testing, as well as by-subject and by-item random intercepts and random slopes for all relevant main effects. We will exclude random correlations this model. The 95% confidence intervals will be calculated for all estimates (using the *broom* package and *Wald* method in RStudio [118]). In addition, we will account for small imbalances in numbers of the predictors’ levels (due to participants not completing all aspects of the test) by entering all predictors in mean-centered form (ie, deviation coding). All entered predictors will be checked for collinearity (using the *cor* function and model output in RStudio). Lastly, we will use post-hoc likelihood-ratio (*X*^2^) model comparisons to quantify the predictive power and exact significance level of all initially significant or trending effects (ie, *P*<.1) revealed by the generalized linear mixed-effect models analyses, as well as the Akaike information criterion. The analyses will include consideration of potential moderators or confounders (ie, presenting gender, age, education). Where significant differences are observed within the primary dependent variables, post-hoc comparisons between groups and level of impairment will calculate unbiased effect sizes, their exact 95% confidence intervals, and Bayes factors [120]. Along with the confidence intervals, a Bayes factor analysis will allow us to quantify the strength of the evidence in support of the null hypothesis if no difference between groups exists. Due to a lack of previous research using Bayes factors in this area, we will use an uninformed prior for the Bayes analysis with a Cauchy width of 0.7.

**Table 2:** Variables, measures, and methods of analyses.

Variable or outcome		Hypothesis	Outcome measure(s)	Method(s) of analysis
**Primary**				
	Global cognitive function	Improves outcomes at 6 months, maintained at 12 months	Montreal Cognitive Assessment	Mixed effect models, post-hoc analyses, effect sizes, Bayes factors
	Reading speed	Improves outcomes at 6 months, maintained at 12 months	Minnesota Low Vision Reading Test, International Reading Speed Test	Mixed effect models, post-hoc analyses, effect sizes, Bayes factors
	Reading comprehension	Improves outcomes at 6 months, maintained at 12 months	Comprehension questions of International Reading Speed Test	Mixed effect models, post-hoc analyses, effect sizes, Bayes factors
	Perceived effort	Improves outcomes at 6 months, maintained at 12 months	Reading Habit Questionnaire	Mixed effect models, post-hoc analyses, effect sizes, Bayes factors
**Secondary**				
	Reading acuity	Improves outcomes at 6 months, maintained at 12 months	Minnesota Low Vision Reading Test	Mixed effect models, post-hoc analyses, effect sizes, Bayes factors
	Critical print size	Improves outcomes at 6 months, maintained at 12 months	Minnesota Low Vision Reading Test	Mixed effect models, post-hoc analyses, effect sizes, Bayes factors
	Reading accuracy	Improves outcomes at 6 months, maintained at 12 months	Semantic judgment task	Mixed effect models, post-hoc analyses, effect sizes, Bayes factors
	Subjective reports of changes in reading habits	Improves outcomes at 6 months, maintained at 12 months	Reading Habit Questionnaire	Mixed effect models, post-hoc analyses, effect sizes, Bayes factors
	Episodic learning	Improves outcomes at 6 months, maintained at 12 months	Rey Auditory Verbal Learning Test	Mixed effect models, post-hoc analyses, effect sizes, Bayes factors
	Memory encoding, storage, and retrieval	Improves outcomes at 6 months, maintained at 12 months	Rey Auditory Verbal Learning Test	Mixed effect models, post-hoc analyses, effect sizes, Bayes factors
	Attention, speed, and mental flexibility	Improves outcomes at 6 months, maintained at 12 months	Oral Trail Making Test	Mixed effect models, post-hoc analyses, effect sizes, Bayes factors
	Semantic fluency	Improves outcomes at 6 months, maintained at 12 months	Word Fluency: FAS & animal naming	Mixed effect models, post-hoc analyses, effect sizes, Bayes factors
	Correlations among sensory and cognitive variables	Improved outcomes are positively correlated	All measures	Pearson and Spearman correlation coefficients
**Tertiary**				
	Demographic variables	Influence the cognitive benefits of reading rehabilitation, with varied directionality	Clinical chart review	Covariate in mixed effect models
	Hearing impairment severity	Increase in hearing impairment reduces cognitive benefit of reading rehabilitation	Audiogram, Canadian Digit Triplet Test, Hearing Questionnaire	Covariate in mixed effect models
	Mental health	Decrease in mental health reduces cognitive benefit of reading rehabilitation	Depression, Anxiety and Stress Scale	Covariate in mixed effect models

#### Exploratory Analyses

It is likely that some participants will self-select not to complete the follow-up or to abandon parts of the recommended intervention tools and techniques. Therefore, we will examine differences in participant characteristics and consider intent-to-treat within the statistical analyses. These analyses will also explore whether the heterogeneity of the sample has possible effects on the outcomes. We are currently exploring additional funding possibilities in order to extend the study period, should the recruitment of a larger sample be required. This increase in available data would also address possible limitations in statistical power for the large number of potential variables to be included in the analyses.

These data may allow us to follow potential exploratory avenues of analysis. First, if the recruited participants naturally divide into groups with different degrees of change in reading (eg, individuals whose self-reported reading effort was reduced or whose reading activity increased versus those for whom we do not observe a change in reading behavior), we can compare these 2 groups directly, while still considering factors such as hearing status or presenting gender. Second, if no such clear division occurs but participants naturally distribute along a continuum of reading effort and reading behavior variables, we can simply use the secondary reading outcome measure(s) as predictors to test whether any or all of the reading effort measures emerge as significant covariates and examine whether scores on the primary cognitive outcome measures improved over time across our 3 groups, after removing the effect of rehabilitation on reading. Third, if the amount and type of data allow, we will use latent factor analysis to explore whether specific clusters of variables are specifically associated with improvements in reading ability or improved performance on cognitive tests.

In line with the requirements of our funding agencies, both sex and gender were considered during study planning [121]. The present study will not include any biological measures of sex; however, with regards to presenting gender, we will pay specific attention to the binary men:women ratio in the sample as recruitment will likely result in a larger number of women who will participate, given their increased lifespan and larger numbers in vision rehabilitation settings [78]. In addition, gender differences in openness to the acceptance and use of assistive technology have been reported [122-124], which can directly affect the potential benefit of reading rehabilitation approaches that include electronic magnification (eg, the use of an iPad [50,51]). Therefore, presenting gender will feature prominently in our statistical analyses.

### Research Ethics Approval

The Comité d'éthique de la recherche of the Centre de recherché interdisciplinaire en readaptation du Montreal metropolitain (CRIR#1284-1217) has provided institutional review board approval. This committee is responsible for research protocols involving recruitment from and testing on the sites of the local clinical partners for this study. The principal investigator will obtain renewal of the ethics approval annually. We submitted all aspects of the study design and data analysis as a stage 1 pre-registered report (ClinicalTrials.gov ID: NCT04276610; February 19, 2020).

### Protocol Amendments

Any necessary changes to the protocol (eg, safety and security measures necessary due to COVID-19) are planned in collaboration with the partner sites in order to adhere to the requirements of the Quebec Ministry of Health. These updates and amendments are the submitted for approval to the Comité d'éthique de la recherche of the Centre de recherché interdisciplinaire en readaptation du Montreal metropolitain.

### Consent

One of the 4 trained research assistants will obtain written informed consent at the first in-person meeting, either in the home or at the lab in the partner sites. Consent forms are available in large print in both English and French. Participants are free to abandon the study at any time, and this choice will not affect their care at the study partner sites.

### Confidentiality

All electronic study data will be stored in a password-protected Excel file on an encrypted server at the Université de Montréal and can only be accessed through a password-protected computer in a locked lab space. All records that contain personal identifiers, such as consent forms and questionnaires, will be stored separately from study records. Each participant file will be identified by code number. Study data will not be released to anyone outside the research team.

### Access to Data

Data access is limited to the members of the research team and their trainees. In order to ensure confidentiality, the data that will be available to the research team members will only contain deidentified information.

### Ancillary and Posttrial Care

All participants will be rehabilitation clients of 1 of the 2 partner sites. Therefore, they will have access to all available service and referral pathways that are part of the care offer. Should the research team suspect or a participant express a potential need for services or referral (eg, counseling), the research team will connect the participant with their respective rehabilitation file manager at the partner site for follow-up.

### Dissemination Policy

All members of the research team have the right to access and analyze all data, for the purpose of dissemination. The principal investigator will oversee dissemination activities in order to ensure that team members with the necessary topic expertise are involved in each aspect of dissemination. Study results will be made available in open-access format whenever possible and will be presented in formats that are accessible to researchers, clinicians, policy makers, members of the public, participants, and all other stakeholders. The study also distinguishes itself with its multifaceted approach to integrated knowledge translation and dissemination. The clinical partners are represented on the research team, directly influencing the study and maintaining the clinical relevance of the study goals. They are part of a local network of rehabilitation research sites, the Center for Interdisciplinary Rehabilitation Research of Greater Montreal [125], providing the research team a unique opportunity to disseminate their knowledge translation activities that are planned to occur after the study is completed. As an extension of this network, established collaborations with the Sense-Cog group in Europe [126], Envision University in the United States [127], and Canadian Consortium on Neurodegeneration in Aging knowledge translation team [128] provide access to sensory-cognitive-specific knowledge translation opportunities that will facilitate the distribution and implementation of our findings. Furthermore, Dr. Swenor, as a visually impaired scientist [129] and team member, brings a unique and important knowledge translation, equity, and inclusion perspective to the study. Finally, all collaborators and partners will participate in the development of a Canadian Institutes of Health Research Café scientifique [130] at study completion. This type of public, open science dissemination event is designed to disseminate the results at a clinical level and inform stakeholders as well as participants and their friends and family of the study outcomes

## Results

The Fonds de recherche Quebec - Santé & Fondation Turmel funded the first 2 years of this peer-reviewed protocol (see Multimedia Appendix 1). The Canadian Institutes of Health Research provided a Project Grant within a Patient-Oriented Research Priority Announcement for a third year. We submitted all aspects of the study design and data analysis as a stage 1 pre-registered report (ClinicalTrials.gov ID: NCT04276610; February 19, 2020). The Comité d'éthique de la recherche of the Centre de recherché interdisciplinaire en readaptation du Montreal metropolitain (CRIR#1284-1217) has provided institutional review board approval. This committee is responsible for research protocols involving recruitment from the clinical partners (local sensory rehabilitation centers) within this study: the Centre de réadaptation Lethbridge-Layton-Mackay du Centre intégré universitaire de santé et de services sociaux du Centre-Ouest- de-l’Île-de-Montréal and the Institut Nazareth et Louis-Braille du Centre intégré de santé et de services sociaux de la Montérégie-Centre. Recruitment began on April 24, 2019. As of March 13, 2020, 38 low vision participants and 7 control participants had been enrolled. Recruitment was paused due to lock-down on March 13, 2020 and will recommence once the COVID-19 crisis has passed to a point where face-to-face data collection with older adults becomes feasible again.

## Discussion

To our knowledge, this study protocol is the first to propose the exploration of the potentially beneficial effect of reading rehabilitation on cognitive functioning in older adults with AMD. Both vision and hearing impairments were recently specifically mentioned as variables that should be considered in the assessment of cognitive functioning [5]. Therefore, given the current trends in the global aging of the population and the emerging importance of sensory health for cognitive health [3], the timing of this study is optimal in order to elucidate whether reading rehabilitation may be able to reduce the potential risks that vision impairment poses for declines in cognitive functioning.

## Appendix

Multimedia Appendix 1 Peer Review Comments by the Fonds de recherche Quebec - Santé (funding for Year 1 and 2) and Canadian Institutes of Health Research (year 3) committees.

## Abbreviations

AD: Alzheimer’s disease
AMD: age-related macular degeneration
dB HL: decibel hearing level
DSI: dual sensory impairment
MMSE: Mini-Mental State Examination
MoCA: Montreal Cognitive Assessment
PTA: pure-tone average
RAVLT: Rey Auditory Verbal Learning Test
