# Supplementary material for: Effect of Reading Rehabilitation for Age-Related Macular Degeneration on Cognitive Functioning: Protocol for a Nonrandomized Pre-Post Intervention Study
Source: JMIR Res Protoc. 2021 Mar 11;10(3):e19931. doi: 10.2196/19931 (PMC7995070; doi:10.2196/19931)
Supplement: Multimedia Appendix 1 [file resprot_v10i3e19931_app1.pdf]

ÉVALUATION DES DEMANDES

No de dossier :	252235
Candidat :	**Walter Wittich**
Titre du projet :	Les mots et le cerveau : Est-ce que la réadaptation en lecture pour les personnes ayant une dégénérescence maculaire liée à l'âge peut améliorer le fonctionnement cognitif ?<br><br>Words on the Brain: Can Reading Rehabilitation for Age-Related Macular Degeneration Improve Cognitive Functioning?

SHORT SUMMARY:

This proposal explores the link between sensory deficits and dementia. There is an accepted link between hearing and cognitive function in the elderly. However, the role of decline in visual function in reduced cognitive function is less explored. The applicants hypothesize that improving reading ability in AMD patients will improve cognitive function. To test their hypothesis, they propose to study changes in cognitive ability between two groups of AMD patients: one that receives reading rehabilitation and a control group consisting of patients receiving vision rehab not directed towards reading.

STRENGTHS:

- This is a quite unique and interesting proposal
- The application addresses a significant and understudied area of research
- There are many potential benefits for AMD patients
- This is a high quality multidisciplinary team with strong expertise in their respective areas of research. There is also a well-established history of collaboration between the team members.

POINTS OF DISCUSSION:

- The major concern raised by members of the committee is that the control group might not be appropriate
- There are some concerns as well about the hypothesis and the proposed effects of vision rehab on cognition
- The team may benefit from consultation with a biostatistician
- The applicants should address the possibility of crossover
- Several concerns about methodology

BUDGET:

- Budget is fine, adequate.

Consensus	3,5
Pointage final :	3,58
Rang :	1

**Review Type / Type d'évaluation:**	Reviewer 1 / Évaluateur 1
**Name of Applicant / Nom du chercheur:**	Wittich, Walter
**Application No. / Numéro de demande:**	427221
**Agency / Agence:**	CIHR/IRSC
**Competition / Concours:**	Project Grant/Subvention Projet
**Committee / Comité:**	Social Dimensions in Aging/Dimensions sociales du vieillissement
**Title / Titre:**	Words on the Brain: Can Reading Rehabilitation for Age-Related Vision Impairment Improve Cognitive Functioning?

Summary of Application/Résumé de la demande:

Existing research indicates that hearing rehabilitation may have a beneficial effect on cognitive functioning due to a reduced cognitive demand during listening. Pilot work by this research team suggests correlations between sensory and cognitive variables, such that low-vision individuals with better sensory performance have higher cognitive scores, and lower levels of anxiety, stress and depression.

In the present project, the researchers wish to investigate whether improving reading performance by providing magnification tools to individuals with low vision will have a beneficial effect on cognitive functioning. The research questions include: (a) does improved reading performance via reduced reading effort improve memory performance in older adults with age-related vision loss, (b) is this effect equally strong when individuals have both vision and hearing loss, and (c) do changes in reading performance following low-vision rehabilitation modify cortical architecture of the visual system (or more specifically lead to changes in cortical thickness as observed with structural MRI).

The design proposed uses 3 groups, namely (a) 75 older adults with acquired low vision acuity due to glaucoma or macular degeneration), (b) 75 older adults with both vision and hearing loss, and (c) 50 age-matched controls. The researchers will use structural MRI scans with a subsample from these groups.

Participants will be followed over a 12 month period, and will be tested at intake, and again 6 and 12 months after the intervention.

The intervention consists of receiving services from partner centers regulated by the Quebec Ministry of health, and includes (a) prescription of appropriate glasses for near and distance glasses, (b) optical or screen content magnification devices, and (c) optimal lighting assessment in the home.

**Review Type / Type d'évaluation:**	Reviewer 1 / Évaluateur 1
**Name of Applicant / Nom du chercheur:**	Wittich, Walter
**Application No. / Numéro de demande:**	427221
**Agency / Agence:**	CIHR/IRSC
**Competition / Concours:**	Project Grant/Subvention Projet
**Committee / Comité:**	Social Dimensions in Aging/Dimensions sociales du vieillissement
**Title / Titre:**	Words on the Brain: Can Reading Rehabilitation for Age-Related Vision Impairment Improve Cognitive Functioning?

Strengths and Weaknesses/Forces et faiblesses:

Strengths:

This is a strong team with an excellent network of collaborators. The fundamental question of the links between sensory input and cognitive performance in older adults is a very interesting one, and these researchers propose an innovative way of exploring this.

They describe an extensive set of variables (e.g., anxiety, reading variables, cognitive variables, neurological measures) that will provide a thorough measure of the impact of the intervention. The development of measures adapted to vision loss is an important asset, as is the plan to collect measures that will allow comparisons to the Canadian Longitudinal Study on Aging

Weaknesses:

My main concern with the methods proposed is that the intervention to be used seems weak and relatively ill controlled. The clinics offer a variety of services, and no manipulation check of which participants received which aids seems to be planned. More importantly, the researchers suggest that the services provided to each client are based at least partly on the clients' own goals and objectives --- it seems likely that clients with different levels of cognitive performance (and different levels of interest in reading) will request different levels of support.

A related issue is that there are no controls in terms of how long since the participant has been unable to read comfortably. This seems important: if reading provides intellectual stimulation, then the amount of time the person was without this stimulation (or was willing to go without this stimulation) may be an important factor.

Another concern is not excluding participants from the study on the basis of dementia/ cognitive performance. The researchers comment on the advantage of being able to explore the magnitude of the manipulation effect across different cognitive starting points. However, they do not discuss how many individuals within this population can be expected to have dementia, and whether sample sizes will be sufficient to explore this issue. Also, the researchers do not discuss how analyses will be carried out if there are different proportions of individuals with dementia across the three groups. Finally, will individuals with Alzheimer's disease or other forms of dementia be excluded from the MRI analysis? If so, how will the exclusion criteria be defined?

**Review Type / Type d'évaluation:**	Reviewer 1 / Évaluateur 1
**Name of Applicant / Nom du chercheur:**	Wittich, Walter
**Application No. / Numéro de demande:**	427221
**Agency / Agence:**	CIHR/IRSC
**Competition / Concours:**	Project Grant/Subvention Projet
**Committee / Comité:**	Social Dimensions in Aging/Dimensions sociales du vieillissement
**Title / Titre:**	Words on the Brain: Can Reading Rehabilitation for Age-Related Vision Impairment Improve Cognitive Functioning?

Budget Recommendation/Recommandation budgétaire:

Accepted as presented.

**Review Type / Type d'évaluation:**	Reviewer 1 / Évaluateur 1
**Name of Applicant / Nom du chercheur:**	Wittich, Walter
**Application No. / Numéro de demande:**	427221
**Agency / Agence:**	CIHR/IRSC
**Competition / Concours:**	Project Grant/Subvention Projet
**Committee / Comité:**	Social Dimensions in Aging/Dimensions sociales du vieillissement
**Title / Titre:**	Words on the Brain: Can Reading Rehabilitation for Age-Related Vision Impairment Improve Cognitive Functioning?

Sex and/or Gender Considerations/Notions de sexe et/ou de genre:

Findings from existing research regarding sex and gender are noted, and self-reported sex and gender are included in planned analyses.

**Review Type / Type d'évaluation:**	Reviewer 2 / Évaluateur 2
**Name of Applicant / Nom du chercheur:**	Wittich, Walter
**Application No. / Numéro de demande:**	427221
**Agency / Agence:**	CIHR/IRSC
**Competition / Concours:**	Project Grant/Subvention Projet
**Committee / Comité:**	Social Dimensions in Aging/Dimensions sociales du vieillissement
**Title / Titre:**	Words on the Brain: Can Reading Rehabilitation for Age-Related Vision Impairment Improve Cognitive Functioning?

Summary of Application/Résumé de la demande:

Existing evidence suggests a link between sensory impairment and cognitive decline. Much of this work is informed by the relationship between hearing loss in midlife and dementia risk as well as the efficacy of hearing rehabilitation. This study will examine the efficacy of improving visual function (through reading rehabilitation) for cognitive health. It will address the following research questions:

1)Does reduced reading effort improve memory in older adults with age-related vision loss?
2)Is the effect of reading effort equally strong in individuals who are have visual impairment only?
3)What is the relationship between age-related visual impairment at baseline, and changes in reading performance following low-vision rehab, and changes in the cortical imaging data?

The quasi-experimental design will involve three groups of older adults: 1) low vision, 2) dual sensory impairment (vision and hearing), and 3) age-matched controls. Pre- and post- measures (e.g., cognition, psychosocial, and clinical) will be examined as well as an imaging substudy to examine cortical activity.

**Review Type / Type d'évaluation:**	Reviewer 2 / Évaluateur 2
**Name of Applicant / Nom du chercheur:**	Wittich, Walter
**Application No. / Numéro de demande:**	427221
**Agency / Agence:**	CIHR/IRSC
**Competition / Concours:**	Project Grant/Subvention Projet
**Committee / Comité:**	Social Dimensions in Aging/Dimensions sociales du vieillissement
**Title / Titre:**	Words on the Brain: Can Reading Rehabilitation for Age-Related Vision Impairment Improve Cognitive Functioning?

Strengths and Weaknesses/Forces et faiblesses:

Strengths:

-This study brings together some relevant lines of evidence around sensory impairment and cognition. It seeks to address a critical issue around cognitive impairment and aging.

-Project is being led by an ECI with an impressive publication and funding record. Project is supported by senior scholars in the area of cognition and sensory function.

-Publication record features several articles directly related to the proposed project.

-Existing laboratory infrastructure enhances feasibility of the study as does commitment from partners.

Weaknesses:

-While this is an interesting line of research, rationale for the study is largely based on evidence from hearing impairment. The proposed study is a quasi-experimental clinical trial. Given lack of clear evidence around link between vision and cognition, is it too soon for a trial of this magnitude?

-While the pilot data are intriguing (n=17), the correlations reported are taken from baseline. Reading seems to be associated with MOCA scores (overall measure of cognition).

-The power calculations seem a bit thin which is understandable given limited pilot data. Given the variability in the existing power estimates reported in the literature (.2 – 2.5), how can you be sure the effect of the intervention will be so large (d=1.0)? Is the study sufficiently powered to detect minimal clinically significant differences?

-There does not appear to be mention of cognitive status as an inclusion criterion. Will participants be cognitively intact?

-Intervention will be handled by third party whom the researchers trust. Could there be some quality assurance measures that are put into place to assure the fidelity of the intervention.

**Review Type / Type d'évaluation:**	Reviewer 2 / Évaluateur 2
**Name of Applicant / Nom du chercheur:**	Wittich, Walter
**Application No. / Numéro de demande:**	427221
**Agency / Agence:**	CIHR/IRSC
**Competition / Concours:**	Project Grant/Subvention Projet
**Committee / Comité:**	Social Dimensions in Aging/Dimensions sociales du vieillissement
**Title / Titre:**	Words on the Brain: Can Reading Rehabilitation for Age-Related Vision Impairment Improve Cognitive Functioning?

Budget Recommendation/Recommandation budgétaire:

No budget concerns.

**Review Type / Type d'évaluation:**	Reviewer 2 / Évaluateur 2
**Name of Applicant / Nom du chercheur:**	Wittich, Walter
**Application No. / Numéro de demande:**	427221
**Agency / Agence:**	CIHR/IRSC
**Competition / Concours:**	Project Grant/Subvention Projet
**Committee / Comité:**	Social Dimensions in Aging/Dimensions sociales du vieillissement
**Title / Titre:**	Words on the Brain: Can Reading Rehabilitation for Age-Related Vision Impairment Improve Cognitive Functioning?

Sex and/or Gender Considerations/Notions de sexe et/ou de genre:

Sex and gender considerations adequately addressed.

**Review Type / Type d'évaluation:**	Reviewer 3 / Évaluateur 3
**Name of Applicant / Nom du chercheur:**	Wittich, Walter
**Application No. / Numéro de demande:**	427221
**Agency / Agence:**	CIHR/IRSC
**Competition / Concours:**	Project Grant/Subvention Projet
**Committee / Comité:**	Social Dimensions in Aging/Dimensions sociales du vieillissement
**Title / Titre:**	Words on the Brain: Can Reading Rehabilitation for Age-Related Vision Impairment Improve Cognitive Functioning?

Summary of Application/Résumé de la demande:

Hearing loss having been identified as a potentially modifiable risk factor for age-related cognitive impairment which may be improved through hearing rehabilitation, this study seeks to explore whether visual rehabilitation following vision loss may have similar cognitive benefits. Research questions are as follows:

1.Does improved reading performance associated with reduced cognitive demand during reading improve cognitive function related to memory in older adults with age-related macular degeneration?

2.Is the effect equally strong in people with vision loss alone, compared to people with dual vision and hearing loss?

3.Does visionary rehabilitation improve cortical architecture in people with AMD?

AMD and Alzheimer’s disease derive from the same part of the central nervous system. Inflammation has been identified as causal in both diseases. Retinal drusen and plaques in the brain both contain amyloid beta and other inflammatory modulators. Causal pathway hypothesised is that low vision makes reading harder cognitive work, that vision rehabilitation would reduce this cognitive burden resulting in improved cognitive performance. The study is a prospective, pre-post, quasi-experimental design involving three patient groups (50 with AMD vision loss, 50 with AMD vision loss and hearing loss, 50 normal controls) assessed at three time points (baseline, 6 months, 12 months). The first two groups enter the standard programme of visual rehabilitation funded by the Quebec Health Insurance programme in two Montreal sites. Controls receive no intervention. A substantial battery of cognitive, visual, hearing and general health status measurement instruments are applied at each data point. In addition, subsamples of 15 participants from each group undergo MRI scanning to assess cortical thickness.

**Review Type / Type d'évaluation:**	Reviewer 3 / Évaluateur 3
**Name of Applicant / Nom du chercheur:**	Wittich, Walter
**Application No. / Numéro de demande:**	427221
**Agency / Agence:**	CIHR/IRSC
**Competition / Concours:**	Project Grant/Subvention Projet
**Committee / Comité:**	Social Dimensions in Aging/Dimensions sociales du vieillissement
**Title / Titre:**	Words on the Brain: Can Reading Rehabilitation for Age-Related Vision Impairment Improve Cognitive Functioning?

Strengths and Weaknesses/Forces et faiblesses:

Strengths

This is a well-designed study given the circumstances of its subjects and the nature of their health conditions. The topic is potentially very important in terms of understanding the development of dementia and potential risk factor modification and subsequent disease prevention. The team has substantial experience and are undertaking a pilot study now which appears to show the study is at least feasible as far as the first wave of data collection is concerned. Eligible patients are already attending for vision rehabilitation so the study in the main fits into clinical operational processes fairly easily. Data collection is comprehensive. The use of MRI to study whether there are measurable changes in cortical structures associated with the intervention will help tease out the relationship between the intervention and outcomes in terms of whether what appears to be cognitive improvement in fact derives from a learned 'skill' (ie easier reading) rather than from actual cognitive change.

Weaknesses

The lack of randomisation and a direct control group is an obvious problem, but it is difficult to think how such a design would be feasible. A wait-list control group, whose access to the intervention was delayed by, say, up to a year, would probably deter patient participation even if REBs supported it and some patients were prepared to consent. The battery of instrumentation looks very time consuming from the patients' perspective. The applicants do not mention typical completion times. Their previous work suggests 10% attrition between data collection points, but the pilot study, as described, doesn't present data from the second or third data points and the actual rate may be more than the quoted figure.

**Review Type / Type d'évaluation:**	Reviewer 3 / Évaluateur 3
**Name of Applicant / Nom du chercheur:**	Wittich, Walter
**Application No. / Numéro de demande:**	427221
**Agency / Agence:**	CIHR/IRSC
**Competition / Concours:**	Project Grant/Subvention Projet
**Committee / Comité:**	Social Dimensions in Aging/Dimensions sociales du vieillissement
**Title / Titre:**	Words on the Brain: Can Reading Rehabilitation for Age-Related Vision Impairment Improve Cognitive Functioning?

Budget Recommendation/Recommandation budgétaire:

Very reasonable given the intensity of data collection. It may require patient and caregiver remuneration for time spent on it: the idea that people are sitting around with nothing to do just waiting for researchers to turn up on the doorstep and entertain them is a little dated. Even for the elderly with AMD.

**Review Type / Type d'évaluation:**	Reviewer 3 / Évaluateur 3
**Name of Applicant / Nom du chercheur:**	Wittich, Walter
**Application No. / Numéro de demande:**	427221
**Agency / Agence:**	CIHR/IRSC
**Competition / Concours:**	Project Grant/Subvention Projet
**Committee / Comité:**	Social Dimensions in Aging/Dimensions sociales du vieillissement
**Title / Titre:**	Words on the Brain: Can Reading Rehabilitation for Age-Related Vision Impairment Improve Cognitive Functioning?

Sex and/or Gender Considerations/Notions de sexe et/ou de genre:

Review Type/Type d'évaluation: SO Notes /Notes de l'agent scientifique
Name of Applicant/Nom du chercheur: Wittich, Walter
Application No./Numéro de demande: 427221
Agency/Agence: CIHR/IRSC
Competition/Concours: 2019-09-11 Project Grant/Subvention Projet
Committee/Comité: Social Dimensions in Aging/Dimensions sociales du vieillissement
Title/Titre: Words on the Brain: Can Reading Rehabilitation for Age-Related Vision Impairment Improve Cognitive Functioning?

Assessment/Évaluation:

Strengths:

The application is from a strong team with an established network. The applicants plan on including a large set of variables, including measures of vision loss. They propose an interesting use of MRI technology, and their proposal would enable comparisons with CLSA data. Theirs is definitely an interesting idea, using an innovative approach; it is clearly valuable to look at the link between vision and cognitive impairment. The study findings could be important, and there was considerable enthusiasm for the proposal among committee members. However, there were several concerns raised.

Weaknesses:

As a starting point, members of the committee felt the application could be strengthened with more pilot data. For example, the time required for data collection is unclear. In their consideration of the sample size required, the applicants may wish to provide more detailed sample size calculations, taking into account attrition, and how they would deal with sample heterogeneity such as the presence of individuals with cognitive impairment, and differences in the duration of reduced visual (and hearing) stimulation prior to the intervention. It is also unclear how the applicants would deal with participants who have dementia for the MRI components. Regarding the intervention, its standardization was unclear given that services may be based on clients’ own goals and expectations, and questions remained regarding its intensity and fidelity.

Budget:

Appropriate.

Review Type/Type d'évaluation: SO Notes /Notes de l'agent scientifique
Name of Applicant/Nom du chercheur: Wittich, Walter
Application No./Numéro de demande: 427221
Agency/Agence: CIHR/IRSC
Competition/Concours: 2019-09-11 Project Grant/Subvention Projet
Committee/Comité: Social Dimensions in Aging/Dimensions sociales du vieillissement
Title/Titre: Words on the Brain: Can Reading Rehabilitation for Age-Related Vision Impairment Improve Cognitive Functioning?

Assessment/Évaluation:

****

Note: The final rating of the application, provided in the Notice of Decision (NOD), is the averaged rating of the peer review committee members following the discussion of the application during the committee meeting, and therefore may differ from the ratings provided by the assigned reviewers in their respective reviews.

Remarque : La cote définitive de la demande, qui apparaît dans l'avis de décision, représente la moyenne des cotes accordées par les membres du comité d' évaluation par les pairs après avoir débattu de la demande à la réunion du comité. Elle peut donc différer de celle donnée par les évaluateurs dans leur évaluation respective.
